# The Battle for Iron between Bacterial Pathogens and Their Vertebrate Hosts

**DOI:** 10.1371/journal.ppat.1000949

**Published:** 2010-08-12

**Authors:** Eric P. Skaar

**Affiliations:** Department of Microbiology and Immunology, Vanderbilt University Medical Center, Nashville, Tennessee, United States of America; University of California San Francisco, United States of America

Iron is a vital nutrient for virtually all forms of life. The requirement for iron is based on its role in cellular processes ranging from energy generation and DNA replication to oxygen transport and protection against oxidative stress. Bacterial pathogens are not exempt from this iron requirement, as these organisms must acquire iron within their vertebrate hosts in order to replicate and cause disease.

## Vertebrates Sequester Iron from Invading Pathogens

**Figure 1:**
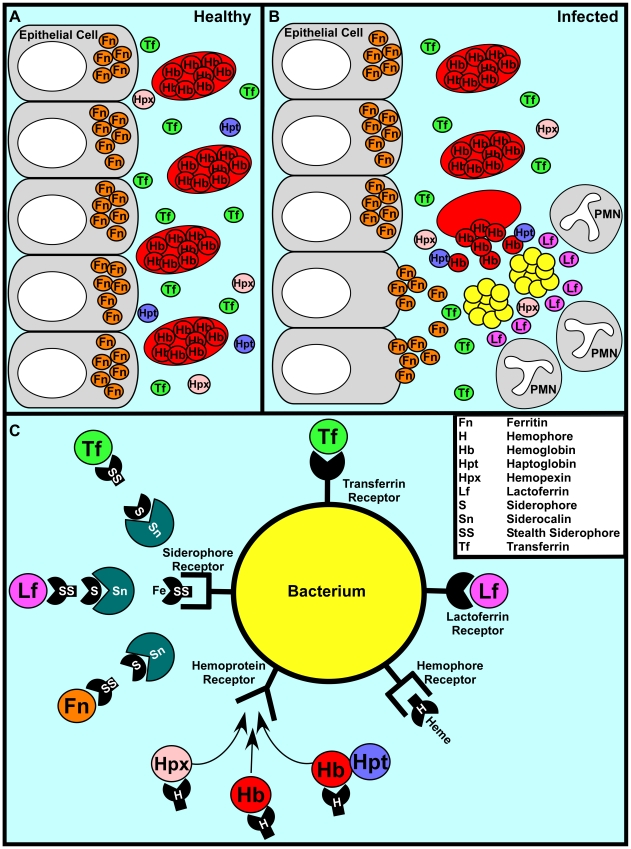
A representative battle during the war for iron. (A) In a healthy individual iron is largely intracellular, sequestered within ferritin or as a cofactor of heme complexed to hemoglobin within erythrocytes. Any extracellular free iron is rapidly bound by circulating transferrin. Hemoglobin or heme that is released as a result of natural erythrocyte lysis is captured by haptoglobin and hemopexin, respectively. Taken together, these factors ensure that vertebrate tissue is virtually devoid of free iron. (B) During infection, bacterial pathogens are capable of altering the battlefield to increase the abundance of potential iron sources. Bacterial cytotoxins damage host cells, leading to the release of ferritin while hemolytic toxins lyse erythrocytes, liberating hemoglobin. The resulting inflammatory response includes the release of lactoferrin from secondary granules contained with polymorphonuclear leukocytes (PMNs). Bacterial pathogens are capable of exploiting these diverse iron sources through the elaboration of a variety of iron acquisition systems. (C) Bacterial pathogens can acquire iron through receptor-mediated recognition of transferrin, lactoferrin, hemopexin, hemoglobin, or hemoglobin–haptoglobin complexes. Alternatively, secreted siderophores can remove iron from transferrin, lactoferrin, or ferritin, whereupon siderophore–iron complexes are recognized by cognate receptors at the bacterial surface. Analogously, secreted hemophores can remove heme from hemoglobin or hemopexin and deliver heme to bacterial cells through binding with hemophore receptors. Siderophore-mediated iron acquisition is inhibited by the innate immune protein siderocalin, which binds siderophores and prevents receptor recognition. This host defense is circumvented through the production of stealth siderophores that are modified in such a way as to prevent siderocalin binding.

One of the first lines of defense against bacterial infection is the withholding of nutrients to prevent bacterial outgrowth in a process termed nutritional immunity. The most significant form of nutritional immunity is the sequestration of nutrient iron [1]. The vast majority of vertebrate iron is intracellular, sequestered within the iron storage protein ferritin or complexed within the porphyrin ring of heme as a cofactor of hemoglobin or myoglobin. Further, the aerobic environment and neutral pH of serum ensures that extracellular iron is insoluble and hence difficult to access by invading pathogens. This difficulty is enhanced by the serum protein transferrin, which binds iron with an association constant of approximately 10^36^ [2]. Taken together, these factors ensure that the amount of free iron available to invading bacteria is vastly less than what is required to replicate and cause disease (Figure 1A).

The importance of nutritional immunity as it pertains to iron is exemplified by the increased susceptibility to infection of individuals with iron overload due to thalassemia and primary hemochromatosis, two of the most common genetic diseases of humans [3]. The degree to which transferrin is iron saturated can vary from 25% to 30% in a healthy individual to 100% in patients with hemochromatosis, negating the antimicrobial properties of transferrin-mediated iron sequestration [2]. The impact of this iron overload is perhaps best demonstrated by the enhanced susceptibility of hemochromatosis patients to *Vibrio vulnificus* infections [4]. Whereas *V. vulnificus* is killed by normal blood and rarely causes infection in healthy individuals, it grows rapidly in blood from patients with hemochromatosis, leading to a high risk of fatal infections in this cohort [4]. Moreover, the administration of excess iron increases the virulence of numerous pathogens in animal models, further highlighting the protection provided by nutritional immunity [2], [5].

## Many Bacterial Pathogens Sense Iron Depletion as a Signal That They Are within a Vertebrate Host

Vertebrates are devoid of free iron, ensuring that all bacterial pathogens encounter a period of iron starvation upon entering their hosts. In keeping with this, bacterial pathogens have evolved to sense iron depletion as a marker of vertebrate tissue. This sensing typically involves transcriptional control mediated by the iron-dependent repressor known as Fur (ferric uptake regulator) [6]. Fur binds to target sequences in the promoters of iron-regulated genes and represses their expression in the presence of iron. In the absence of iron, Fur-mediated repression is lifted and the genes are transcribed. Fur orthologs have been identified in numerous genera from both Gram-negative and Gram-positive bacteria and contribute to the virulence of both animal and plant pathogens [7].

A number of genes encoding for proteins involved in iron utilization have been reported to be positively regulated by Fur during iron-replete conditions [8]. This positive regulation occurs through Fur-mediated repression of a small RNA that represses genes encoding iron utilization proteins. This second level of regulation prevents the use of iron by non-essential enzymes during times of iron starvation. RNA-dependent regulation of iron utilization is a conserved process that has been identified in multiple bacterial pathogens, including *Vibrio* sp., *Pseudomonas aeruginosa*, *Escherichia coli*, *Shigella flexneri*, and *Bacillus subtilis* [8].

Many high G+C content Gram-positive bacteria express an additional iron-dependent repressor belonging to the DtxR family. The DtxR family was named for its founding member, the diphtheria toxin repressor. In fact, one of the first iron-dependent virulence factors described was diphtheria toxin produced by *Corynebacterium diphtheria* [2]. DtxR family members negatively regulate genes involved in processes ranging from iron acquisition to virulence factor expression [5].

In addition to sensing alterations in iron levels, bacterial pathogens can also sense heme as a marker of vertebrate tissue. Heme-responsive activators have been identified in *Serratia marcescens*, the pathogenic *Bordetella*, *C. diphtheriae*, *Bacillus anthracis*, and *Staphylococcus aureus* [5], [9], [10], [11]. Heme-sensing systems presumably alert bacterial pathogens when they are in contact with vertebrate tissues rich in heme, triggering the expression of systems involved in heme-iron acquisition and metabolism.

## All Bacterial Pathogens Can Circumvent Iron Withholding

In order to thrive within vertebrates, bacteria must possess mechanisms to evade nutritional immunity. Perhaps the most elegant mechanism to circumvent iron withholding is employed by *Borrelia burgdorferi*, the causative agent of Lyme disease. *B. burgdorferi* has evolved to not require iron for growth by substituting manganese in its metal-requiring enzymes [12]. Most pathogens have not evolved this simple defense strategy and instead circumvent iron withholding through high-affinity iron uptake mechanisms that compete against host-mediated sequestration. These uptake systems can be divided into three main categories: siderophore-based systems, heme acquisition systems, and transferrin/lactoferrin receptors (Figure 1C).

Siderophores are low molecular weight iron-binding complexes that are secreted from bacteria. Siderophores bind iron with an association constant that can exceed 10^50^, enabling bacteria to compete with iron sequestration by transferrin and lactoferrin [2]. Upon removing iron from host proteins, iron-loaded siderophores are bound by cognate receptors expressed at the bacterial surface. The siderophore–iron complex is then internalized into the bacterium and the iron is released for use as a nutrient source. The importance of siderophores to bacterial virulence is demonstrated by the decreased fitness of siderophore-defective strains in animal models of infection [2], [7].

Heme acquisition systems typically involve surface receptors that recognize either heme or heme bound to hemoproteins such as hemoglobin or hemopexin. Heme is then removed from hemoproteins and transported through the envelope of bacteria into the cytoplasm. Once inside the cytoplasm, the iron is released from heme through the action of heme oxygenases or reverse ferrochelatase activity [13]–[15]. Bacterial pathogens can also elaborate secreted heme-scavenging molecules that remove heme from host hemoproteins. These molecules, known as hemophores, are functionally analogous to siderophores but are proteins that target heme, whereas siderophores are small molecules that target iron atoms [16]. As is the case with siderophore transport systems, genetic defects in heme acquisition systems reduce bacterial fitness in many animal models of infection [2], [7].

In addition to acquiring iron from transferrin and lactoferrin through siderophore-based mechanisms, some bacteria are capable of direct recognition of these host proteins. The most well-studied transferrin and lactoferrin receptors are present in pathogenic members of the Neisseriaceae and Pasteurellaceae [7]. These proteins are modeled to recognize human transferrin or lactoferrin, leading to iron removal and subsequent transport into the bacterial cytoplasm. Human challenge models with *Neisseria gonorrhoeae* suggest that gonococci expressing both lactoferrin and transferrin receptors exhibit a selective advantage within the host, underscoring the importance of this iron acquisition strategy to these organisms [5], [17].

## Targeting Bacterial Iron Acquisition as a Second Layer of Defense against Infection

A second layer of nutritional immunity employed by vertebrates is to combat siderophore-mediated iron acquisition through the production of siderocalin [18]. Siderocalin, also referred to as lipocalin-2 or neutrophil gelatinase-associated lipocalin (NGAL), is a protein that is secreted by neutrophils in response to infection. Siderocalin binds enterobactin, the primary siderophore of many enteric bacteria, and sequesters the siderophore–iron complex, preventing bacterial uptake. Mice lacking siderocalin exhibit increased sensitivity to enterobactin-expressing bacteria, demonstrating the pathophysiological relevance of this anti-siderophore defense system [19].

The requirement for iron by bacterial pathogens ensures that iron acquisition systems are expressed and surface exposed during infection. This fact has established surface-exposed iron receptors as viable vaccine candidates for the prevention of bacterial infection. The enterobactin receptor FetA from *Neisseria meningitidis* [20], the siderophore receptor IroN from *Escherichia coli* [21], the hemoglobin receptor HgbA from *Haemophilus ducreyi* [22], surface proteins of the *S. aureus* Isd heme uptake machinery [23], and a combination of *E. coli* iron acquisition proteins [24] are examples of iron utilization systems that have been proposed as candidate vaccines.

## Bacterial Pathogens Are Leading the Arms Race for Nutrient Iron

Resistance to siderocalin is a conserved strategy across multiple pathogenic microbes. A primary bacterial defense against siderocalin involves the production of stealth siderophores. These molecules represent structurally modified enterobactin-type siderophores that are resistant to siderocalin binding. The Gram-positive pathogen *B. anthracis* produces the siderophore petrobactin, which incorporates a 3,4-dihydroxybenzoyl chelating subunit that prevents siderocalin binding [25]. Similarly, *Salmonella* Typhimurium produces salmochelin, a glycoslyated derivative of enterochelin that is not targeted by siderocalin [26]. The production of stealth siderophores is the most recently uncovered layer in the arms race for nutrient iron during host–pathogen interactions. Undoubtedly, we have not yet discovered the complete armamentarium in this battle that has tremendous implications for the outcome of bacterial infections.
